# Diversity and biogeographical patterns in the diet of the culpeo in South America

**DOI:** 10.1002/ece3.70176

**Published:** 2024-08-13

**Authors:** Jorge Lozano, Marta Guntiñas, Rodrigo Cisneros, Esther Llorente, Adrián Duro, Aurelio F. Malo

**Affiliations:** ^1^ Department of Biodiversity, Ecology and Evolution, Faculty of Biological Sciences Complutense University of Madrid Madrid Spain; ^2^ Department of Biology, Geology, Physics and Inorganic Chemistry. School of Experimental Sciences and Technology King Juan Carlos University Madrid Spain; ^3^ Department of Biological Sciences Universidad Técnica Particular de Loja Loja Ecuador; ^4^ GloCEE Global Change Ecology and Evolution Research Group, Department of Life Sciences University of Alcalá Madrid Spain

**Keywords:** Andean fox, canids, carnivores, *Lycalopex culpaeus*, Neotropical region, top predator, trophic ecology

## Abstract

Here we describe the dietary patterns of the culpeo or Andean fox (*Lycalopex culpaeus*) on a biogeographical scale. We also analyse the influence of exotic lagomorphs on its diet and explore differences between culpeo subspecies. We selected 17 mutually comparable diet studies, which include 19 independent diet assessments. Then, we extracted and standardized the values of the different diet components from these studies and calculated the relative frequency of occurrence of the 10 main trophic groups that we found. Further, we calculated the Shannon‐Wiener *H*′ trophic diversity index. The results showed that small mammals (41%), lagomorphs (21%), invertebrates (12.4%) and large herbivores (7.3%) were the most consumed groups. A factorial analysis of all trophic groups rendered four orthogonal factors that were used as response variables in relation to a set of environmental predictors. Altitude correlated with most factors (i.e. trophic groups). Exotic lagomorphs were consumed in lowlands, in higher latitudes and in regions showing high values of the human footprint index, enriching in those areas the culpeo's trophic spectrum. There were no differences in diet between the two main culpeo subspecies analysed, *L.c. culpaeus* and *L.c. andinus*. Finally, the best explanatory models (general linear model) of trophic diversity selected, using Akaike's information criterion, showed that the most diverse diets were those composed of large herbivores, edentates, carnivorous species, birds and herptiles (i.e. reptiles and frogs). Trophic diversity was low in rainy areas where big rodents dominated the diet. Neither latitude nor altitude seemed to have an effect on the trophic diversity of the culpeos, as they were not retained by the final models.

## Graphical abstract

The most diverse culpeo's diets were those composed of large herbivores, edentates, carnivorous species, birds and herptiles (i.e. amphibians and reptiles). Trophic diversity was low in rainy areas where big rodents dominated the diet.

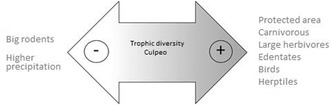

## 1 INTRODUCTION

The functioning of ecosystems is determined and conditioned by a multitude of ecological processes and parameters, such as evolutionary history and the dynamics of species interaction, such as parasitic, competitive or predator–prey relationships, the latter being the most important in the configuration of food webs (Ritchie et al., 2012; Soe et al., 2017). In fact, large carnivores and mesopredator predation tend to play a prominent, and often key, role in the functioning of ecosystems (Newsome & Ripple, 2015), affecting prey population cycles (Kausrud et al., 2008) and producing multiple cascading effects (Coulson & Malo, 2008; Ripple et al., 2014).

In turn, the ecology of a predatory species is influenced by the conditions of the environment in which it lives. Thus, when the different populations that make up a widely distributed species are considered, there is a wide range of environmental conditions that vary across its range. Faced with different ecological conditions each species presents a certain degree of flexibility in their behaviour, which depending on its range and geneflow between subpopulations, can define the species as generalist or specialist. In particular, and in relation to the extent of the trophic niche and prey selection criteria, predators can be characterized as generalists or opportunists, strict specialists or facultative specialists (Glasser, 1982, 1984; Jaksic, 2007).

In the case of predators that have a wide distribution range, strong spatial variation in diet patterns is observed when studied at a biogeographic scale, as shown for numerous species such as the brown bear (*Ursus arctos*) (Vulla et al., 2009), the Eurasian otter (*Lutra lutra*) (Clavero et al., 2003), the European wildcat (*Felis silvestris*) (Lozano et al., 2006), the European badger (*Meles meles*) (Hounsome & Delahay, 2005) or the red fox (*Vulpes vulpes*) (Soe et al., 2017). For example, it is usual to find clear correlations in diet variation with latitude and climate, also related to the trophic plasticity associated with generalist or facultative specialist strategies (Cox & Moore, 2005; Lozano et al., 2006; Virgós & Casanovas, 1999). Indeed, diet can have important implications at several levels. On the one hand, there are a multitude of factors associated with the trophic behaviour of species, which can have repercussions on other aspects of their ecology. For example, the abundance and distribution of native prey not only affect dietary patterns but also space use, morphological characteristics and mating cycles acting at different temporal scales (Dayan & Simberloff, 1996). On the other hand, the incorporation of exotic or non‐native species into the diet and the consumption of anthropogenic food waste can also directly affect the abundance, distribution and behaviour of carnivore species (Bino et al., 2010). In addition, competition among predators and their differential vulnerability can modify ecological processes such as resource use patterns, as seen in medium‐sized carnivores in North America (Lesmeister et al., 2015), as well as evolutionary processes such as physical characteristics of the predator (Jiménez et al., 1996).

To the factors mentioned above, we must add the growing uncertainty on the ability of predators and prey to respond to the effects of global phenomena such as climate change (Bailey & Pol, 2016; Keith & Bull, 2017), and other anthropogenic factors (Sandom et al., 2014), which can promote important changes in trophic interactions at a global scale (Grosbois et al., 2008; Merilä, 2012) triggering profound changes in the structure of ecosystems (Newsome et al., 2015). For this reason, a better understanding of the trophic ecology of species and their interaction with environmental factors at various scales is necessary to develop species conservation strategies that mitigate the effects of global problems on ecosystems.

The way to evaluate the trophic strategy and plasticity of a species is to approach its study at large spatial scales, through the comparison of habitat‐ and region‐specific diets within its range, based on the review and meta‐analysis of local data. For many species, this biogeographical dietary approach is not yet possible due to the few studies available at a local scale. Fortunately, this is not the case for the culpeo (*Lycalopex culpaeus*), also called Andean fox, a canid that is distributed in various habitats from the northern Andes, through Ecuador, Peru, Bolivia, Chile and Argentina (Guntiñas et al., 2021; Lucherini, 2016), for which a number of diet studies have been published. Although on a global scale, the International Union for Conservation of Nature (IUCN) classified the culpeo for its entire range as ‘Least Concern’ (Lucherini, 2016), this good conservation status is not mirrored at regional scales. For instance, in Colombia and Ecuador the culpeo is listed as a threatened species (Lozano et al., 2021), emphasizing the need to deepen the knowledge of its ecology.

Local studies on the culpeo diet interpret the trophic strategies of the species differently (see Guntiñas et al., 2021), considering it a strict carnivore (Iriarte et al., 1989; Jiménez & Novaro, 2004; Redford & Eisenberg, 1992), a practically insectivorous predator (Guzmán‐Sandoval et al., 2007) with tendencies to frugivory (Cornejo & Jiménez, 2001; Ebensperger et al., 1991), or a predator with high level of trophic plasticity that is capable of using a wide combination of resources (Castro et al., 1994; Jaksic et al., 1993; Johnson & Franklin, 1994). Recent studies suggest that culpeo behaves more like a facultative specialist (Guntiñas et al., 2017). However, to date, no studies have been carried out that consider the data as a whole at a biogeographical scale so that the global trophic patterns can be detected, as well as their ecological correlates, which would allow a panoramic view of the culpeo trophic ecology.

The present work reviews culpeo diet studies and data published throughout the species range, with the following aims: (1) to describe the general patterns of the culpeo diet at a biogeographical scale through a meta‐analysis; (2) to explore the environmental factors that determine the culpeo diet variability; (3) to assess the degree of the consumption of exotic lagomorphs by culpeos, particularly of European hare (*Lepus europaeus*) and European rabbit (*Oryctolagus cuniculus*) and the influence of this consumption on the use of native fauna as a trophic resource (Crespo & de Carlo, 1963; Jaksic, 1998; Novaro et al., 2000; Rubio et al., 2013); (4) to evaluate the dietary differences between the two main culpeo subspecies described by Guzmán et al. (2009) and (5) to obtain an explanatory model of the trophic diversity of culpeo in South America.

Overall, we hypothesize that the main trophic groups consumed by culpeos will vary with latitude and altitude, depending on the availability of each prey group to culpeos (Guntiñas et al., 2021). We also test the hypothesis that the presence of exotic lagomorphs substitutes native fauna in the diet of culpeos, showing a reduced consumption of other main trophic groups (e.g. Crespo & de Carlo, 1963; Novaro et al., 2000). Similarly, the same effect would be expected if culpeos included plant matter in their diet (e.g. Ebensperger et al., 1991). Furthermore, if morphological differences among culpeo subspecies were correlated with diet (Guntiñas et al., 2021; Guzmán et al., 2009; Guzmán‐Sandoval et al., 2007), we would expect to find significant differences in the consumption of prey groups by the different subspecies. Finally, we hypothesize that trophic diversity values will increase from northern areas to mid‐latitudes, corresponding to a higher availability of trophic resources (e.g. Clavero et al., 2003; Lozano et al., 2006).

## 2 MATERIALS AND METHODS

We carried out a complete compilation of the published articles and other reports on the diet of the species. For information published prior to 1988, we used the review by Medel and Jaksic (1988). For works published after 1988, we carried out a systematic bibliographic search using the ‘Web of Knowledge’, ‘Google Scholar’ and ‘Scopus’ servers, including terms such as diet, culpeo, Andean fox, *Lycalopex culpaeus*, páramo wolf and also the specific names that have been used to describe the culpeo previously (i.e. *Dusicyon culpaeus* and *Pseudalopex culpaeus*) (see for more details, Guntiñas et al., 2021).

A subset of the studies found was chosen to be used for statistical analyses, which met a number of requirements to ensure the representativeness of the data and the statistical power (Guntiñas et al., 2021). Firstly, they had to provide tables of data on the culpeo diet in the form of either frequency of occurrence, relative frequency of occurrence or number of prey items, as well as the sample sizes for the scats or stomachs analyzed. The data had to describe all the resources consumed by the species, so that studies with data focusing only on a particular prey group were excluded. Secondly, the papers had to have a minimum sample size of 30 scats and/or stomachs (Lozano et al., 2006; Soe et al., 2017) per habitat type considered, treating these habitat units as independent samples. Finally, data had to be representative of the annual cycle in each habitat unit (Lozano et al., 2006).

**FIGURE 1:**
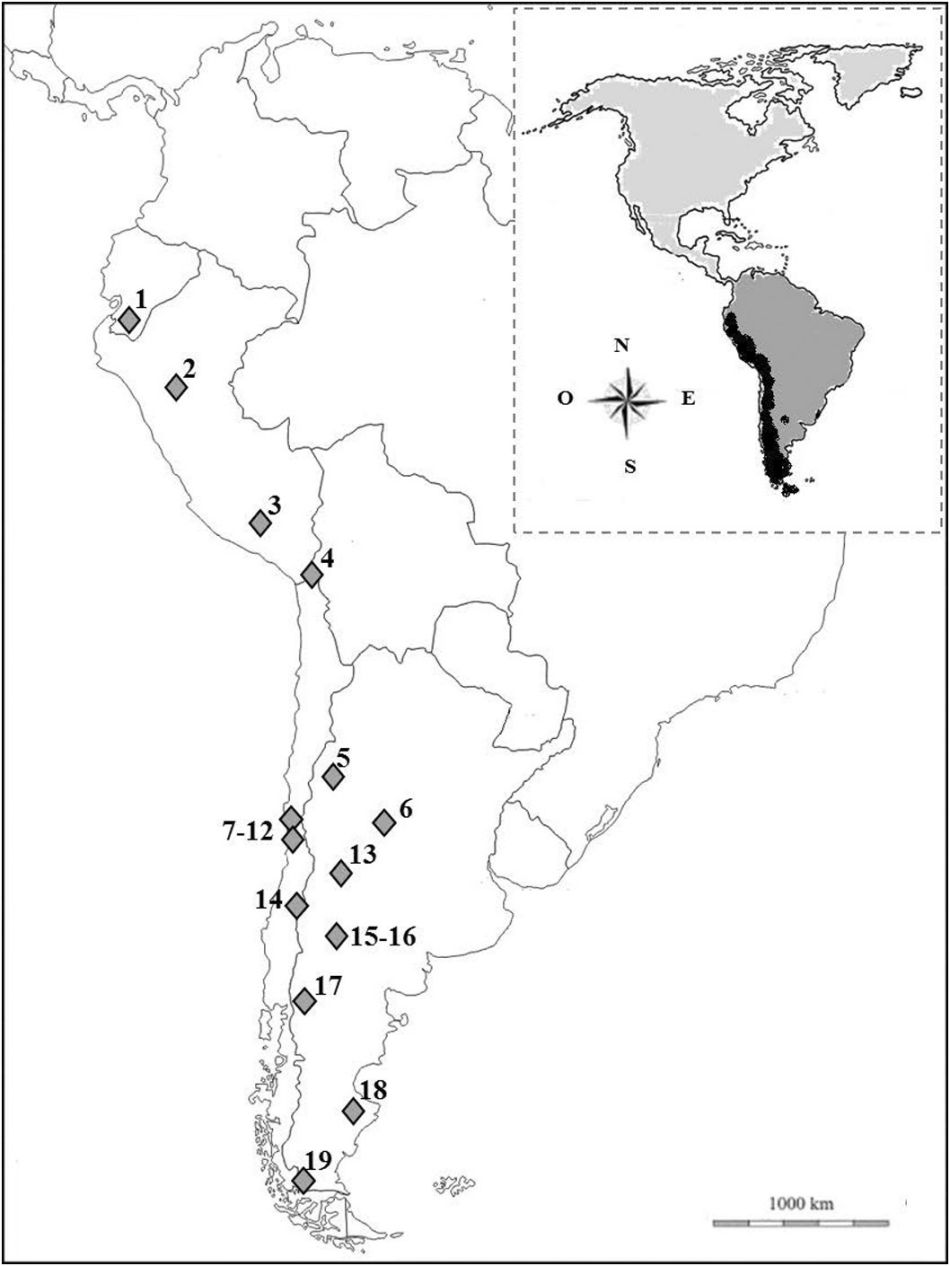
Culpeo's range in America (small box) and location of the different study areas: 1. Guntiñas et al. (2017); 2. Romo (1995); 3. Cornejo and Jiménez (2001); 4. Marquet et al. (1993); 5. Walker et al. (2007); 6. Pía et al. (2003); 7. Ebensperger et al. (1991); 8. and 9. Iriarte et al. (1989); 10. Jaksic et al. (1980); 11. and 12. Rubio et al. (2013); 13. Berg (2007); 14. Achilles (2007); 15. Palacios et al. (2012); 16. Novaro et al. (2000); 17. Monteverde and Piudo (2011); 18. Zapata et al. (2005); 19. Johnson and Franklin (1994).

Of all, 17 (16 articles and one thesis) studies met the aforementioned criteria, producing 19 independent samples (Figure 1). Altogether the data set included 4115 scats and/or stomachs with a total of 6220 trophic elements. Due to the variability in data presentation between studies, we standardized the data of the selected studies by calculating the relative frequency (RF) of occurrence for each independent sample. For the analyses, we did not consider whether identified trophic items came from stomachs or scats given that it is not usual to find a bias in the origin of the analyzed material (e.g. Lozano et al., 2006).

### 2.1 Selection of variables

We categorized the different elements that composed the culpeo diet into the same 10 trophic groups used previously by Guntiñas et al. (2021): ‘small mammals’ (i.e. any mammal weighing less than 300 g, mainly rodents, insectivores and some marsupial species), ‘big rodents’ (i.e. rodent species weighing more than 300 g), ‘lagomorphs’, ‘carnivorous’ (i.e. carnivores and carnivorous marsupials weighing more than 300 g), ‘edentates’ (i.e. armadillos); ‘large herbivores’ (i.e. deer, camelids and livestock), ‘birds’, ‘eggs’, ‘herptiles’ (i.e. reptiles and frogs) and ‘invertebrates’. The RF of occurrence for each of these trophic groups was recalculated by dividing the number of items in each group by the total number of items. Given that plant matter in the diet cannot be calculated into RF values only its presence for each study was recorded.

We also calculated the trophic diversity of the culpeo in each independent sample as the value of the Shannon‐Wiener *H*′ diversity index (Weaver & Shannon, 1949). In order to calculate and compare the value of the index between the different studies, the trophic groups were standardized to the same taxonomic level as the groups in the less detailed publications, leaving the following 19 groups: rodents, soricids, procyonids, edentates, mustelids, marsupials, lagomorphs, livestock (i.e. sheep), cervids, camelids, felids, carrion, birds, reptiles, fish, amphibians, invertebrates, eggs and rubbish. Thus, these groups allowed us to calculate an *H*′ index as accurate as possible.

We recorded the main habitat type in which the work was carried out using the geographical location of each of the studies where the samples have been taken. Further, we determined whether the site was part of a protected area or not, the average annual rainfall, the altitude and the latitude. We also measured the degree of interference of human activity in the territory by calculating the human footprint index (Sanderson et al., 2002) for each independent sample. This index is a combination of human population density, land alteration and human infrastructures (such as power lines and roads), ranging between 0 (no human interference) and 100 (maximum human interference). We used two human footprint index layers downloaded for the years 1993 and 2009 from the Center for International Earth Science Information Network (CIESIN) and, based on the sampling year for each study, we attributed the value of the index layer corresponding to the nearest year to each study area. On the other hand, we tested whether introduced (i.e. exotic) lagomorph species existed in the different study areas. For this purpose, the layers of presence of the species of interest available on the IUCN server were downloaded and the overlap with the sampling areas of the selected studies was checked. To observe possible differences in diet according to the subspecies, each study attributed the subspecies *L.c. andinus*, *L.c. culpaeus* or *L.c. reissii* based on their geographical distribution (Guzmán et al., 2009). Although other subspecies have been also proposed, only these ones are currently recognized by all authors (Guntiñas et al., 2021). The subspecies *L.c. reissii* was finally represented by one paper only, so that it was not considered in the analyses.

### 2.2 Statistical analysis

In order to perform parametric analyses, we first checked both the normality of the considered variables and the homogeneity of variances through Levene's test, transforming the variables that were not normal (Zar, 2009). Alternatively, we checked whether the kurtosis of these variables was positive, which allows us to assume a low probability of committing type I statistical error (Underwood, 1996).

We characterized the different types of culpeo diet by grouping the RFs of the 10 main trophic groups into orthogonal factors carrying out a factor analysis and using the principal component analysis (PCA) algorithm. We also tested for spatial autocorrelation in the values of the extracted factors and the trophic diversity values (*H*′) by calculating Moran's index *I* and their respective correlograms (Dormann et al., 2007; Rangel et al., 2010).

Pearson's correlation between orthogonal factors and latitude, altitude, human footprint index and trophic diversity was calculated. In addition, we checked whether the latter correlated with each of the RFs of the trophic groups. We also explored whether the degree of protection of the study areas (with or without legal protection) and habitat type (scrub, steppe or mosaic) explained variation in the trophic factors. The influence of the presence of plant matter in the diet on trophic factors and the trophic diversity was also tested by performing a two‐way and a one‐way MANOVA analyses, respectively. We also assessed whether the presence of exotic lagomorphs, introduced into the study areas, influenced the diet of the species through a MANOVA with the other nine main groups. Furthermore, we analysed the differences between the diets of the subspecies *L.c. andinus* and *L.c. culpaeus* by means of a MANOVA with the subspecies as a fixed factor and the trophic factors as dependent variables, as well as an ANOVA with the trophic diversity also as a dependent variable.

Finally, general linear models (GLM) were constructed for trophic diversity (*H*′) as a response variable, using 10 predictor variables: the four trophic orthogonal factors, latitude, altitude, protected area, habitat type, precipitation and the human footprint index. Of the total number of models obtained, the most parsimonious were identified through a selection process applying the Akaike's criterion (Burnham & Anderson, 2002). Software for conducting the statistical analyses included SAM v.4.0 (Rangel et al., 2010) and Statistica 10 (StatSoft Inc, 2011).

## 3 RESULTS

**FIGURE 2:**
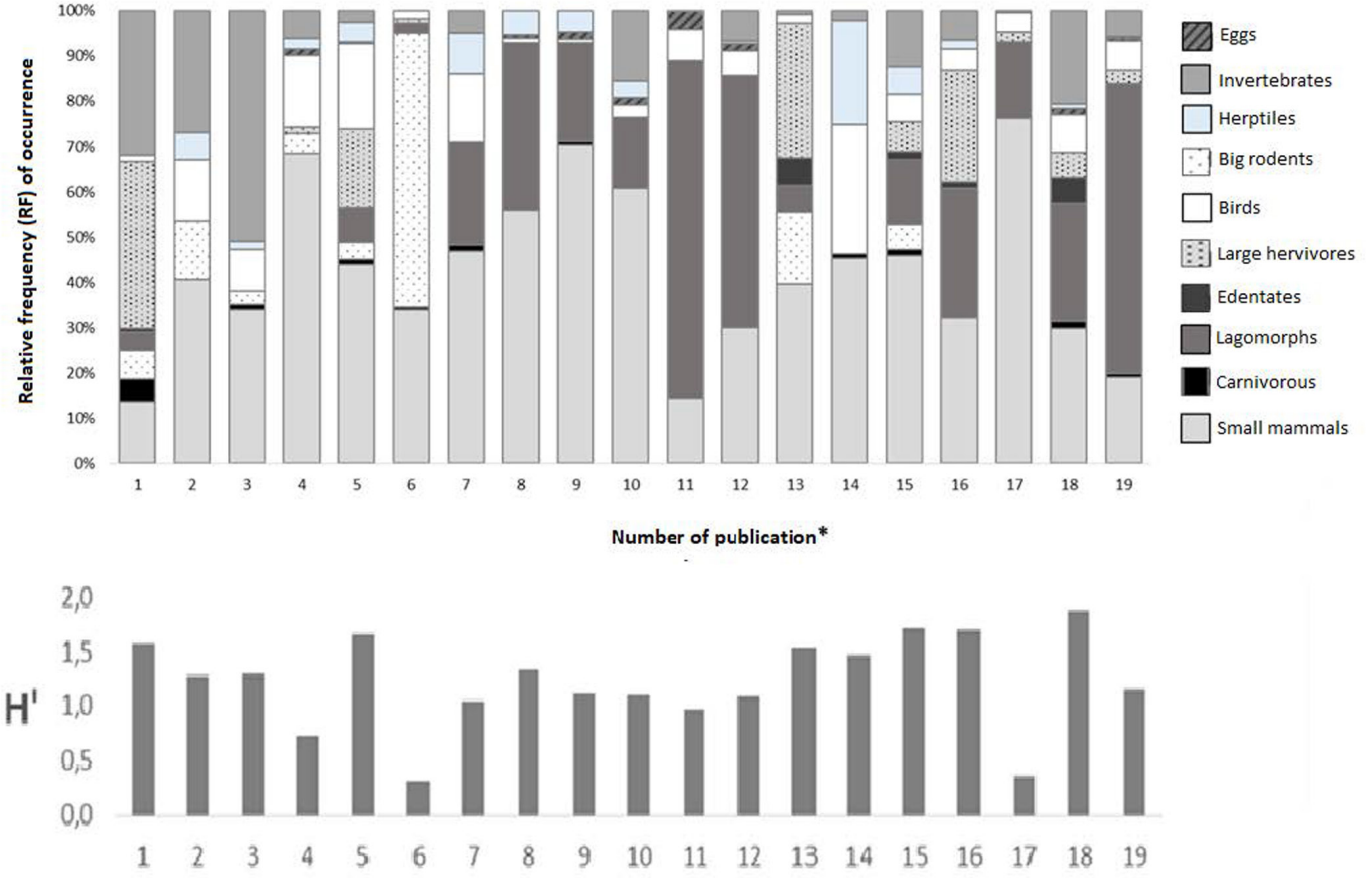
Relative frequency (RF) of occurrence of the 10 trophic groups and values of Shannon‐Wiener trophic diversity (*H*′) index for each independent sample considered in this study. *Publication number: 1. Guntiñas et al. (2017); 2. Romo (1995); 3. Cornejo and Jiménez (2001); 4. Marquet et al. (1993); 5. Walker et al. (2007); 6. Pía et al. (2003); 7. Ebensperger et al. (1991); 8.9. Iriarte et al. (1989); 10. Jaksic et al. (1980); 11.12. Rubio et al. (2013); 13. Berg (2007); 14. Achilles (2007); 15. Palacios et al. (2012); 16. Novaro et al. (2000); 17. Monteverde and Piudo (2011); 18. Zapata et al. (2005); 19. Johnson and Franklin (1994).

Relative frequency values of all the trophic groups calculated for each independent sample, as well as the Shannon‐Wiener *H*′ trophic diversity index, are shown in Figure 2. The mean value of the latter was 1.23 (range: 0.36–1.88). The mean values of RF, expressed as a percentage, were 41% small mammals, 21% lagomorphs, 12.4% invertebrates, 7.3% large herbivores, 7.3% birds, 6% big rodents, 2.9% reptiles and frogs (herptiles), 0.9% carnivorous, 0.8% edentates and 0.7% eggs.

The factor analysis using the RF of the 10 trophic groups generated four orthogonal factors (using standardized varimax rotation of axes) that overall explained 73.6% of the variance of the original variables (Table 1). The first factor describes a gradient from culpeo diets with high consumption of carnivorous, large herbivores and edentates (negative scores) towards less rich diets in these groups. The second factor is a gradient from high consumption of lagomorphs and eggs (negative scores) to poorer diets in these trophic groups and richer in big rodents (positive scores). The third factor is a gradient from diets with high consumption of birds and herptiles (negative scores) to low intake of these prey (positive scores). Finally, the fourth factor describes a gradient from diets with high consumption of small mammals (negative scores) to high consumption of invertebrates (positive scores).

**TABLE 1:** Description of the four orthogonal factors obtained from the factor analysis with the 10 trophic groups considered.

Variables	Factor 1	Factor 2	Factor 3	Factor 4
Large herbivores	**−0.92** ^a^	0.16	0.15	−0.01
Big rodents	0.17	**0.65** ^a^	0.60	−0.04
Birds	0.12	0.08	**−0.70** ^a^	−0.03
Carnivorous	**−0.86** ^a^	0.17	0.05	0.01
Edentates	**−0.46** ^a^	−0.08	0.10	0.16
Eggs	0.21	**−0.81** ^a^	0.12	−0.20
Invertebrates	0.14	0.32	−0.43	**0.74** ^a^
Reptiles/Frogs	0.27	0.25	**−0.76** ^a^	0.05
Lagomorphs	0.1	**−0.9** ^a^	0.33	0.13
Small mammals	0.30	0.13	−0.21	**−0.85** ^a^
Eigenvalue	2.08	2.10	1.82	1.36
% Explained variance	**20.82**	**21.00**	**18.18**	**13.57**

^a^ Indicates significant correlations of the original variables with the extracted factors (*p* < .05) in bold.

According to the correlograms of the four trophic orthogonal factors and trophic diversity *H*′, which were based on Moran's I index, the diet of the culpeo in South America was not spatially structured, given that values describing its diet in the reviewed studies presented no spatial autocorrelation.

**FIGURE 3:**
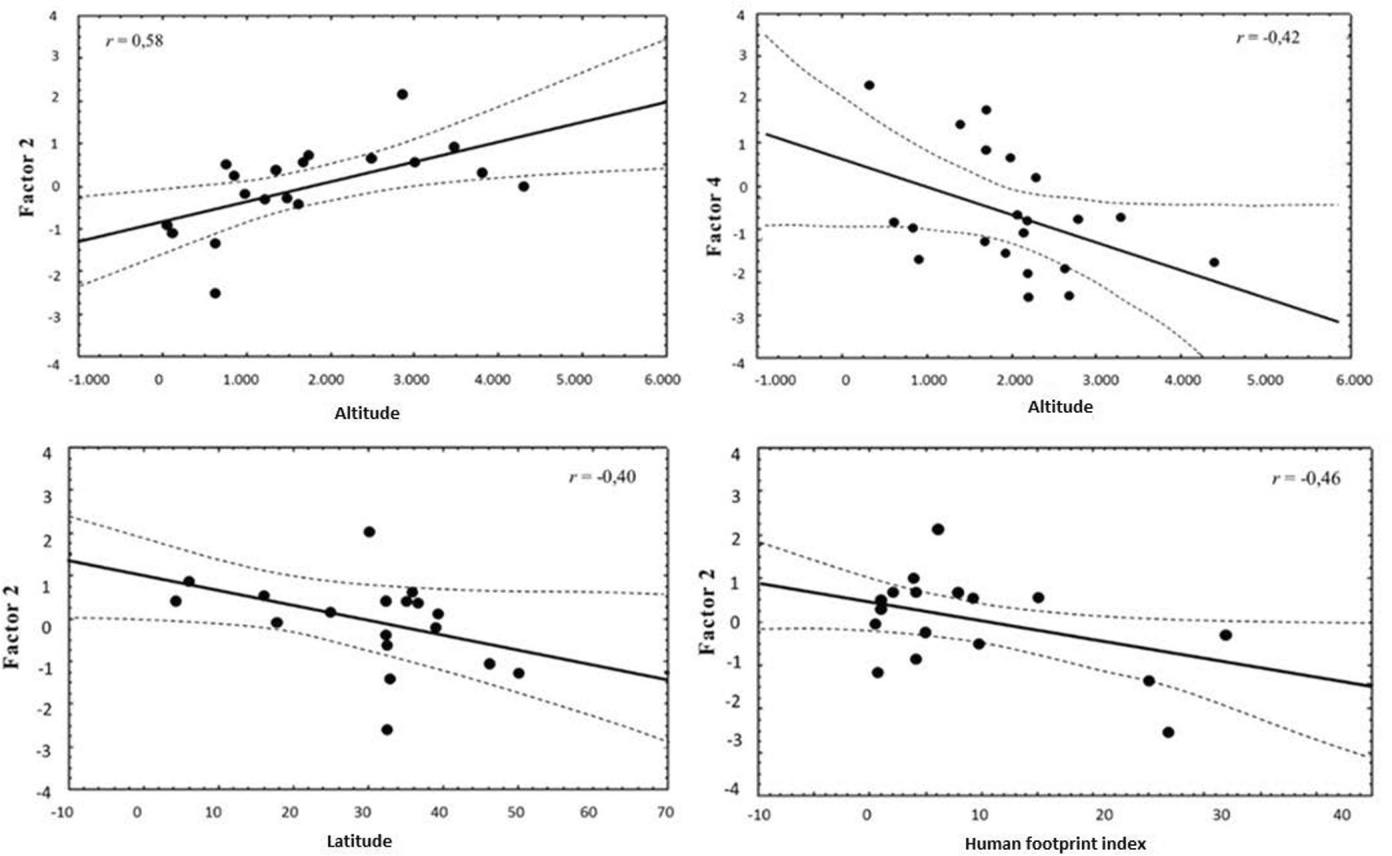
Relationships between factors 2 and 4 with altitude, as well as factor 2 with latitude and the human footprint index.

Factor 1 was negatively correlated with *H*′ trophic diversity, so the inclusion of carnivorous species, large herbivores and edentates in the diet of the culpeo significantly increased the trophic diversity of the canid, as well as the consumption of birds and herptiles, as factor 3 was also negatively correlated with trophic diversity (Table 2). Factor 2 correlated positively with altitude, and negatively with the human footprint index and latitude (Figure 3). Therefore, at higher altitudes (and in areas of lower latitudes and human influence) the culpeo consumes more big rodents, while at higher latitudes and when the human footprint is greater in the environment, consumption of lagomorphs and eggs increases. Factor 4 was negatively correlated with altitude (Figure 3), indicating that higher altitudes result in higher consumption of small mammals and that invertebrates are more prevalent in the diet at lower altitudes. Taking each of the 10 trophic groups one by one, those that contribute the most to the increase in the diversity of the culpeo diet are large herbivores and birds, while diets including big rodents tend to be less diverse (Table 2).

**TABLE 2:** Pearson's correlations of the four trophic orthogonal factors extracted from the factor analysis with latitude, altitude, human footprint index and trophic diversity *H*′ index.

	Latitude	Altitude	Human footprint index	*H*′
Factor 1	0.26	−0.08	0.22	−**0.58** *
Factor 2	−**0.40** ^†^	**0.58** *	−**0.46** *	**0.13**
Factor 3	**0.21**	−**0.22**	**0.19**	−**0.43** *
Factor 4	**0.29**	−**0.43** *	**0.02**	**0.25**
Small mammals				−**0.32**
Carnivorous				**0.33**
Big rodents				−**0.42** ^†^
Lagomorphs				−**0.02**
Edentates				**0.54**
Large herbivores				**0.45** *
Birds				**0.17** *
Eggs				−**0.17**
Herptiles				**0.29**
Invertebrates				**0.16**

*Note*: The correlations between the latter and the relative frequency (RF) of the 10 trophic groups considered in the review (*n* = 19) are also shown.

* Significant correlation (*p* < .05).

^†^ Marginally non‐significant correlation (*p* < .10).

The consumption of plant material tended to influence the diet of the culpeo (MANOVA, Wilks' Lambda = 0.59; *F*_4.14_ = 2.4; *p* = .09), with significant differences in factor 3 (*F*_1.17_ = 4.8; *p* = .04). Thus, when there was consumption of plants, there was also a greater consumption of birds and herptiles. However, the consumption of plant matter had no effect on the trophic diversity *H*′ index (*F*_1.17_ = 0.01; *p* = .98).

**FIGURE 4:**
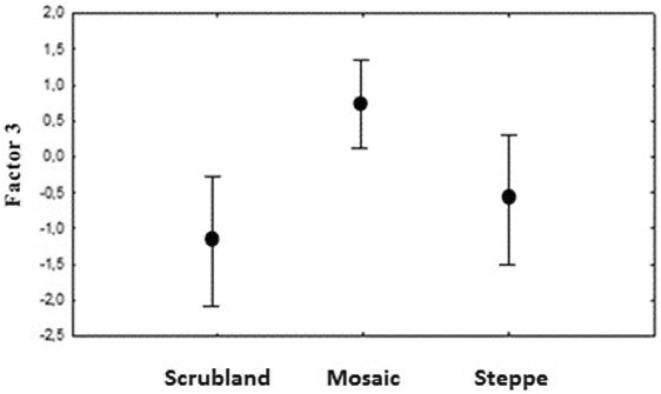
Relationship between factor 3 and habitat type: Scrubland, mosaic and steppe (bars correspond to one standard deviation).

Habitat type influenced the diet of the culpeo given that significant differences were found in the trophic orthogonal factors (Table 3), specifically a positive relationship between factor 3 and the mosaic type habitat (*F*_2.13_ = 8.13; *p* = .005). Therefore, in mosaic habitats the culpeo consumed less birds and herptiles (Figure 4). No significant differences were detected in the trophic factors depending on whether the studies were carried out in protected or unprotected areas (Table 3).

**TABLE 3:** Two‐way MANOVA with the four trophic orthogonal factors as dependent variables and two fixed factors (protected area and habitat type).

	Wilks' lambda	*F*	Df effect	Df error	*p*
Protected area	0.63	1.49	4	10	.28
Habitat type	0.25	2.53	8	20	.04
Interaction	0.5	1.05	8	20	.43

The presence of non‐native (i.e. exotic) lagomorphs in the environment had no significant influence on the diet of the canid (MANOVA, Wilks' Lambda = 0.31; *F*_9.9_ = 2.03; *p* = .153). Furthermore, differences between subspecies *L.c. andinus* and *L.c. culpaeus* were not found in their diet, either considering the trophic orthogonal factors (MANOVA, Wilks' Lambda = 0.37; *F*_4.13_ = 1.52; *p* = .25) or their respective *H*′ trophic diversity index (ANOVA, *F*_1.17_ = 0.03; *p* = .85).

**FIGURE 5:**
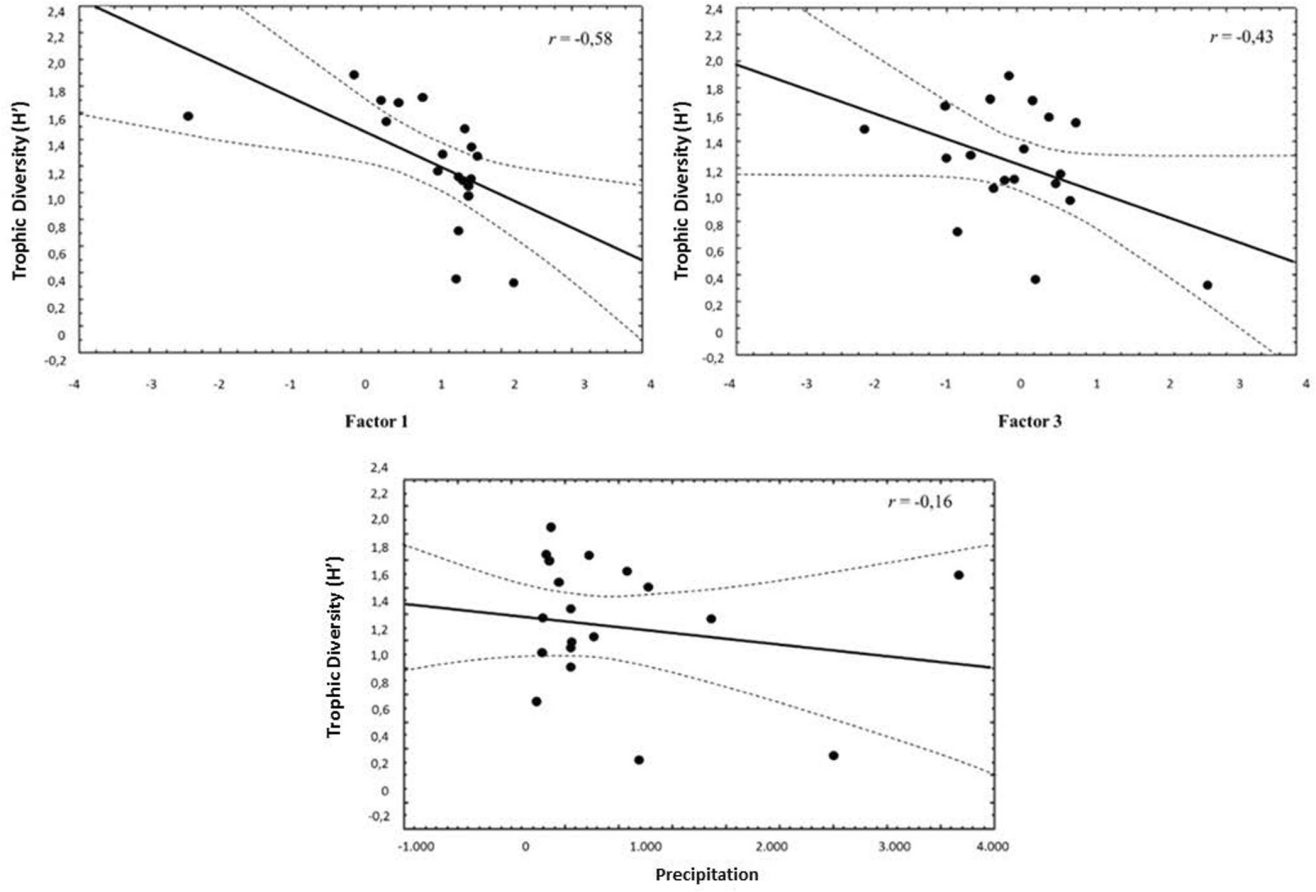
Relationship between trophic diversity (*H*′) index and factors 1 and 3, and precipitation, according to the most parsimonious general linear model obtained.

In total, 1023 GLM were generated for the trophic diversity (*H*′) as a response variable using the 10 predictors mentioned above. By applying the Akaike's selection criterion, only two models were found to be likely (Table 4): the most parsimonious (*r* ^2^ = .75) incorporated factors 1 and 3 in addition to precipitation, while the next model (*r* ^2^ = .79) also incorporated the protected area. In both models, the factors and rainfall were negatively correlated with the *H*′ trophic diversity index (Table 5), so that the most diverse culpeo diets were associated with areas of low rainfall and the RF of carnivorous species, large herbivores, edentates, birds and herptiles in the diet (Figure 5). Considering the second most parsimonious model, culpeos that live in protected areas also could show a greater diversity in their diet than those living in non‐protected areas.

**TABLE 4:** Explanatory models of culpeo's trophic diversity (*H*′) index. It is shown the two parsimonious models obtained with the number of variables used (*k*), Akaike's information criterion (AIC) values for small sample sizes (AICc), the difference between each selected model and the best model (ΔAIC) and each model weight (AICc wi). Only really likely models, with ΔAIC < 2, are shown. The models were ordered from the lowest value (best model) to the highest value according to the AICc.

Models	*k*	AICc	∆ AIC	AICc wi
Factor 1 + Factor 3 + Precipitation	3	9.66	0	0.23
Factor 1 + Factor 3 + Precipitation + Protected area	4	11.50	1.8	0.09

**TABLE 5:** Estimates of the most parsimonious general linear model (GLM) were obtained to explain the variation in the trophic diversity (*H*′) index of the culpeo with factor 1, factor 3 and precipitation as predictors.

	Estimate	Standard error	β	Standard error
Intercept	1.42	0.76		
Factor 1	−0.374	0.07	−.865	0.152
Factor 3	−0.156	0.07	−.360	0.131
Precipitation	−0.0003	0.00007	−.565	0.153

## 4 DISCUSSION

The culpeo is a medium‐sized, mainly carnivorous canid (Guntiñas et al., 2017; Novaro, 1997; Redford & Eisenberg, 1992), which can use a wide diversity of prey throughout its distribution range (Guntiñas et al., 2021). It has been described as a polyphagous species (Pía, 2011), as in some regions, it uses trophic resources such as fruits and invertebrates (Cornejo & Jiménez, 2001; Ebensperger et al., 1991; Guzmán‐Sandoval et al., 2007). Thus, due to this varied diet, the culpeo has generally been considered an opportunistic species (Castro et al., 1994; Crespo & de Carlo, 1963; Jaksic et al., 1980; Johnson & Franklin, 1994). To date, no dietary patterns have been described based on geographical variation, except for the exotic lagomorph consumption in the new areas the culpeo has expanded into, where native prey species seem to have been replaced by exotic ones (Guntiñas et al., 2021; Lucherini, 2016).

Our results show that small mammals are an important prey item across the culpeo's range in terms of RF of occurrence, as reflected in numerous studies (Ebensperger et al., 1991; Iriarte et al., 1989; Johnson & Franklin, 1994; Meserve et al., 1987; Novaro et al., 2000; Pía et al., 2003; Walker et al., 2007; Zapata et al., 2005). In studies that quantified prey availability in the environment, a clear dietary selectivity has been repeatedly shown, that is, in the face of a decline in small mammals (especially rodents), the diet did not change as might be expected in a truly opportunistic species (Jaksic et al., 1992; Martínez et al., 1993). In most of the studies reviewed, small mammals have a high RF of occurrence, although presenting a greater consumption at high altitudes. Therefore, the culpeo shows a certain degree of specialization and could have a strong effect on small mammal populations and be a key predator regulating and/or limiting their populations (Guntiñas et al., 2021; Krebs, 2002).

Species are located at different levels within the food web, where top predators would be at the highest positions, potentially consuming species at lower levels, and with few or no other species that predate them (Essington et al., 2005; Essington & Hansson, 2004). In this context, the pattern of diets rich in carnivorous species, large herbivores and edentates obtained in this review (associated with factor 1), clearly corresponds to those of an apical predator. In Andean systems, the only natural species that can prey on culpeos is the puma (*Puma concolor*) (see Guntiñas et al., 2021). Aside from the puma, the culpeo, also by preying on other carnivores, would act as an important regulator of mammal populations of species of equal or greater size (such as ungulates). This corresponds more to the role of a large carnivore than to those of small or medium‐sized carnivores that the culpeo is classified into (Carbone et al., 2007), and indicates the key role of culpeos in high‐Andean ecosystems (Guntiñas et al., 2021).

Regarding whether the presence of exotic lagomorphs introduced into the environment (i.e. European rabbits and hares) cause a change in the diet of the culpeo, the results of this review indicate that, at the global scale, where these lagomorphs are present, there is no significant variation in the relative frequency of consumption of the main prey groups. Therefore, no widespread substitution of native prey (especially small rodents) has been detected by the dietary dominance of lagomorphs, as suggested in studies at a more local scale (Crespo & de Carlo, 1963; Jaksic, 1998; Novaro et al., 2000; Rubio et al., 2013), although interestingly in other studies, this pattern was not observed (Meserve et al., 1987). At this scale, it seems that introduced species are simply added by culpeos to their trophic spectrum, thus enriching their diet.

Considering the data globally, in the regions where lagomorphs are consumed, small mammals and other prey groups are still consumed, which were also important in terms of RF of occurrence. It should be noted that the lagomorph prey group is the second most important trophic group considering RF intake. As the majority of these are exotic species (the European hare and the European rabbit), with only one study representing a native lagomorph (*Sylvilagus brasiliensis*; Guntiñas et al., 2017), it is likely that the culpeo has taken advantage of this new environmental resource there where it has appeared, similar to how it takes advantage of the availability of big rodents elsewhere. Furthermore, diets rich in lagomorphs and eggs as well as poorer in big rodents (according to factor 2), occur at low altitudes, low latitudes and in regions with an important human influence on the territory. Therefore, the consumption of lagomorphs, especially European hares (taking into account that they are also found at the highest latitudes), can be associated with lowlands and negatively impacted populations of culpeos, such as agricultural and livestock areas, which may coincide with the works describing some substitution in the diet of native prey by exotic lagomorphs (Crespo & de Carlo, 1963; Jaksic, 1998; Novaro et al., 2000; Rubio et al., 2013).

**FIGURE 6:**
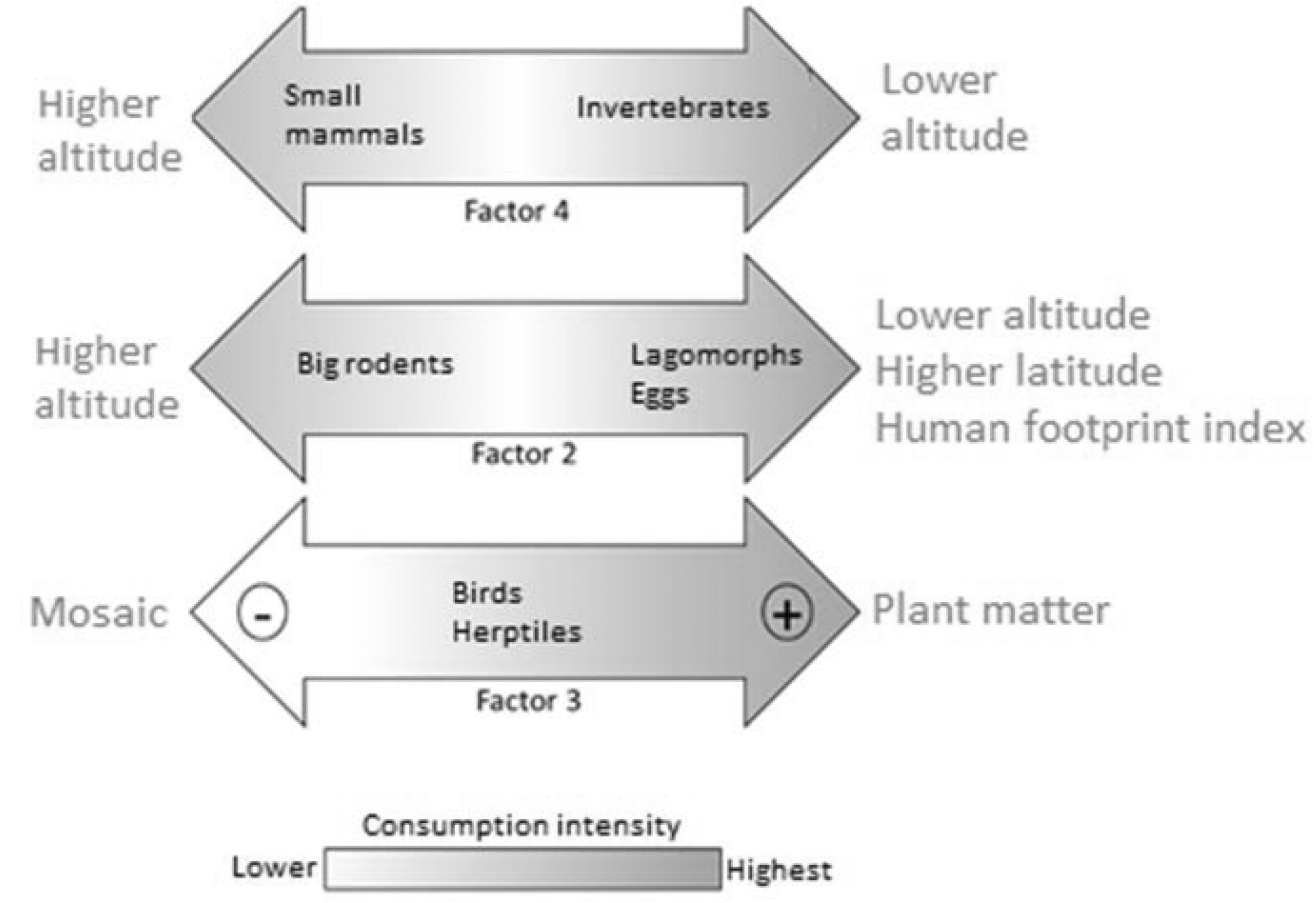
Conceptual model that describes the variation in consumption of trophic groups by the culpeo, grouped in orthogonal factors, in relation to some environmental variables according to the results obtained in this review.

Depending on the altitude, there is a clear pattern of trophic groups consumed, so that in higher altitude areas, there is a greater consumption of small mammals and big rodents, whereas in lower lands, there is a greater consumption of lagomorphs, eggs and invertebrates (Figure 6). In regions of low altitude, the high consumption of invertebrates appears to be consistent with some authors' views on the importance of this group in highly seasonal Mediterranean ecosystems (Correa & Roa, 2005; Ebensperger et al., 1991). On the other hand, at high altitudes, the higher consumption of small mammals and big rodents could perhaps be due to their greater availability, a particular prey selection by culpeos, or also because of the lower availability of other trophic resources.

It seems that the culpeo makes use of the resources available in each study area, determined by a series of environmental factors. Culpeos can specialize in some of them and so not behave merely as a generalist or opportunist. The aforementioned evidence that culpeos can maintain their consumption of small mammals even when their abundance decreases (see also Guntiñas et al., 2021), or the high degree of consumption of large herbivores in certain regions as is the case with ungulates in the high Andes of Ecuador (Guntiñas et al., 2017), indicates that the culpeo behaves more like a facultative specialist than a strict generalist (Glasser, 1984; Guntiñas et al., 2017).

When culpeos consume plant material they often include other food resources, such as birds and herptiles (Figure 6), perhaps also in response to the low abundance or availability of preferred prey (such as rodents), and therefore due to the need to supplement the diet. Nevertheless, the presence of plant matter in the culpeo diet had no effect on the consumption of the other prey groups. In addition, the consumption of birds and herptiles was lower in mosaic‐type habitats, indicating a possible increased availability for the culpeo of this prey in scrubland and steppe areas.

No differences have been found in the diet between the subspecies *L.c. andinus* and *L.c. culpaeus*, neither in the trophic groups consumed nor in the trophic diversity, suggesting that both subspecies display similar trophic behaviour. However, as more diet publications emerge in regions where the ecology of culpeo is poorly known, this picture could change. So, it would be interesting to review the trophic data again in the future, including also the subspecies *L.c. reissii* and increasing the number of case studies of *L.c. andinus*.

**FIGURE 7:**
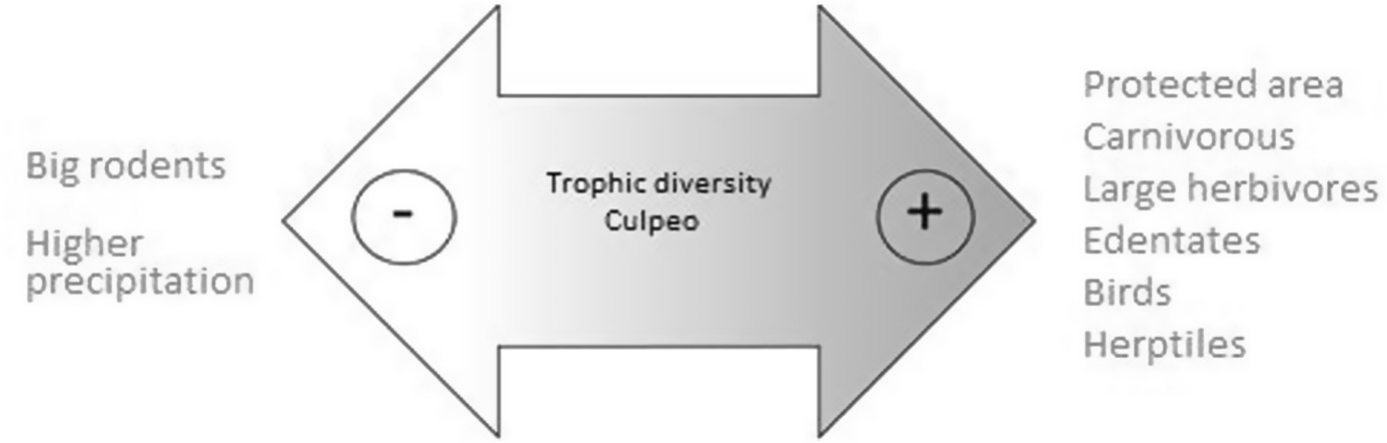
Conceptual model describing the variation in trophic diversity (*H*′) of the culpeo diet according to the results obtained in this review.

On the other hand, one of the best‐known biogeographical patterns of trophic diversity in a number of species is that it is a function of the latitudinal gradient, increasing from the poles towards the tropics (Rosenzweig, 1995). Altitude is also an important driver of species diversity in the diet, as mountains are sites of high diversity and endemism where diets can become more diverse, although under particular conditions, this pattern is not always met (Gentry, 1995). In the case of generalist predators with a wide range of distribution, whose diets reflect the availability of prey in the environment, changes would be expected according to these biogeographical patterns (e.g. Clavero et al., 2003; Lozano et al., 2006). In the case of the culpeo, with populations distributed in a marked latitudinal (from 3° to 50° south latitude) and altitudinal (from sea level to 3800 m) gradient, no differences in trophic diversity were found based on these geographical variables. This result could be explained because the culpeo does not behave like a generalist predator, as mentioned above, so its role as a facultative specialist carnivore (Guntiñas et al., 2017, 2021) could mask latitudinal and altitudinal effects by specializing locally in particular trophic resources. According to the most parsimonious model obtained, the most diverse culpeo diets are those associated with low precipitation rates as well as the consumption of carnivorous species, large herbivores, edentates (i.e. armadillos), birds and herptiles (Figure 7). In addition, it is also possible that in areas with some type of government protection, the canid's diet may be more diverse. Well‐conserved areas, which host populations of species such as large herbivores, carnivores and edentates, would presumably have a greater diversity of species, and therefore of potential prey types, that culpeos could incorporate into their diet.

In conclusion, at the biogeographic scale, the culpeo feeds primarily on small mammals and lagomorphs and specializes facultatively in different types of prey depending on latitude, altitude and habitat. Increased prey availability, for example, in protected areas, may increase the trophic diversity of the species. Finally, there is no evidence that the different subspecies studied have different feeding patterns.

## AUTHOR CONTRIBUTIONS

**Jorge Lozano:** Conceptualization (lead); formal analysis (equal); investigation (lead); methodology (equal); supervision (lead); writing – review and editing (equal). **Marta Guntiñas:** Conceptualization (equal); data curation (equal); formal analysis (equal); investigation (equal); writing – original draft (lead); writing – review and editing (equal). **Rodrigo Cisneros:** Conceptualization (equal); investigation (equal); writing – review and editing (equal). **Esther Llorente:** Data curation (equal); investigation (equal); software (equal); writing – review and editing (equal). **Adrián Duro:** Investigation (equal); resources (equal); software (equal); writing – review and editing (equal). **Aurelio F. Malo:** Investigation (equal); resources (equal); writing – review and editing (equal).

## CONFLICT OF INTEREST STATEMENT

The authors declare that they have no conflict of interest concerning this manuscript.

## Supporting information


Data S1.

## Data Availability

Data set is provided as supplementary files.

## References

[ece370176-bib-0001] Achilles, N. T. (2007). Dieta estival del culpeo (Pseudalopex culpaeus, Molina 1782) en Nevados de Chillán, centro‐sur de Chile . MSc thesis, Facultad de Ciencias Veterinarias, Universidad Austral de Chile.

[ece370176-bib-0002] Bailey, L. D. , & Pol, M. (2016). Tackling extremes: Challenges for ecological and evolutionary research on extreme climatic events. Journal of Animal Ecology, 85(1), 85–89. 26433114 10.1111/1365-2656.12451

[ece370176-bib-0004] Berg, J. E. (2007). The carnivore assemblage of La Payunia reserve, Patagonia, Argentina: Dietary niche, prey availability, and selection . PhD thesis, University of Montana.

[ece370176-bib-0005] Bino, G. , Dolev, A. , Yosha, D. , Guter, A. , King, R. , Saltz, D. , & Kark, S. (2010). Abrupt spatial and numerical responses of overabundant foxes to a reduction in anthropogenic resources. Journal of Applied Ecology, 47, 1262–1271.

[ece370176-bib-0006] Burnham, K. , & Anderson, D. (2002). Model selection and multimodel inference: A practical information‐theoretic approach. Springer‐Verlag.

[ece370176-bib-0007] Carbone, C. , Teacher, A. , & Rowcliffe, J. M. (2007). The costs of Carnivory. PLoS Biology, 5(2), 1–6. 10.1371/journal.pbio.0050022 PMC1769424 17227145

[ece370176-bib-0008] Castro, S. A. , Silva, S. I. , Meserve, P. L. , Gutierrez, J. R. , Contreras, L. C. , & Jaksic, F. M. (1994). Frugivoría y dispersión de semillas de pimiento (*Schinus molle*) por el zorro culpeo (*Pseudalopex culpaeus*) en el Parque Nacional Fray Jorge (IV Región, Chile). Revista Chilena de Historia Natural, 67(2), 169–176.

[ece370176-bib-0009] Clavero, M. , Prenda, J. , & Delibes, M. (2003). Trophic diversity of the otter (*Lutra lutra* L.) in temperate and Mediterranean freshwater habitats. Journal of Biogeography, 30(5), 761–769.

[ece370176-bib-0010] Cornejo, A. , & Jiménez, P. (2001). Dieta del zorro andino *Pseudalopex culpaeus* (Canidae) en el matorral desértico del sur del Perú. Revista de Ecología Latinoamericana, 8, 1–9.

[ece370176-bib-0011] Correa, P. , & Roa, A. (2005). Relaciones tróficas entre *Oncifelis guigna, Lycalopex culpaeus, Lycalopex griseus* y *Tyto alba* en un ambiente fragmentado de la zona central de Chile. Mastozoología Neotropical, 12(1), 57–60.

[ece370176-bib-0012] Coulson, T. , & Malo, A. F. (2008). Case of the absent lemmings. Nature, 456, 43–44. 18987726 10.1038/456043a

[ece370176-bib-0013] Cox, C. B. , & Moore, P. D. (2005). Biogeography: An ecological and evolutionary approach. Blackwell Publishing.

[ece370176-bib-0014] Crespo, J. A. , & de Carlo, J. (1963). Estudio ecológico de una población de zorros colorados *Dusicyon culpaeus* (Molina) en el oeste de la provincia de Neuquén. Revista del Museo Argentino de Ciencias Naturales Bernardina Rivadavia e Instituto Nacional De Investigacion de Ciancias Naturales: Ecologia, 1(1), 1–55.

[ece370176-bib-0015] Dayan, T. , & Simberloff, D. (1996). Patterns of size separation in carnivore communities. In J. Gittleman (Ed.), Carnivore behavior, ecology, and evolution (pp. 243–266). Cornell University Press.

[ece370176-bib-0016] Dormann, C. F. , McPherson, J. M. , Araujo, M. B. , Bivand, R. , Bolliger, J. , Carl, G. , Davies, R. G. , Hirzel, A. , Jetz, W. , Kissling, D. , Kühn, I. , Ohlemüller, R. , Peres‐Neto, P. R. , Reineking, B. , Schroder, B. , Schurr, F. M. , & Wilson, R. (2007). Methods to account for spatial autocorrelation in the analysis of species distributional data: A review. Ecography, 30, 609–628.

[ece370176-bib-0017] Ebensperger, L. A. , Mella, J. E. , & Simonetti, J. A. (1991). Trophic‐niche relationships among *Galictis cuja*, *Dusicyon culpaeus*, and *Tyto alba* in central Chile. Journal of Mammalogy, 72(4), 820–823.

[ece370176-bib-0019] Essington, E. , & Hansson, S. (2004). Predator‐dependent functional responses and interaction strengths in a natural food web. Canadian Journal of Fisheries and Aquatic Sciences, 61, 2215–2226.

[ece370176-bib-0020] Essington, T. E. , Beaudreau, A. H. , & Wiedenmann, J. (2005). Fishing through marine food webs. Proceedings of the National Academy of Sciences of the United States of America, 103(9), 3171–3175. 10.1073/pnas.0510964103 PMC1413903 16481614

[ece370176-bib-0021] Gentry, A. H. (1995). Patterns of diversity and floristic composition in Neotropical montane forests. In S. P. Churchill , H. Balslev , E. Forero , & J. L. Luteyn (Eds.), Biodiversity and conservation of Neotropical montane forests (pp. 103–126). The New York Botanical Garden.

[ece370176-bib-0022] Glasser, J. W. (1982). A theory of trophic strategies: The evolution of faculative specialists. Ecology, 63, 250–262.

[ece370176-bib-0023] Glasser, J. W. (1984). Evolution of efficiencies and strategies of resource exploitation. Ecology, 65, 1570–1578.

[ece370176-bib-0024] Grosbois, V. , Gimenez, O. , Gaillard, J. M. , Pradel, R. , Barbraud, C. , Clobert, J. , & Weimerskirch, H. (2008). Assessing the impact of climate variation on survival in vertebrate populations. Biological Reviews, 83(3), 357–399. 18715402 10.1111/j.1469-185X.2008.00047.x

[ece370176-bib-0025] Guntiñas, M. , Lozano, J. , Cisneros, R. , Llorente, E. , & Malo, A. F. (2021). Ecology of the culpeo (*Lycalopex culpaeus*): A synthesis of existing knowledge. Hystrix, 32(1), 5–17.

[ece370176-bib-0026] Guntiñas, M. , Lozano, J. , Cisneros, R. , Narváez, C. , & Armijos, J. (2017). Feeding ecology of the Andean fox in southern Ecuador: Wild ungulates being the main prey. Contributions to Zoology, 86(2), 169–180.

[ece370176-bib-0027] Guzmán, J. A. , D'Elia, G. , & Ortiz, J. C. (2009). Variación geográfica del zorro *Lycalopex culpaeus* (Mammalia, Canidae) en Chile: implicaciones taxonómicas. Revista de Biología Tropical, 57(1–2), 421–432. 19637719

[ece370176-bib-0028] Guzmán‐Sandoval, J. , Sielfeld, W. , & Ferrú, M. (2007). Dieta de *Lycalopex culpaeus* (Mammalia: Canidae) en el extremo norte de Chile (Región de Tarapacá). Gayana, 71(1), 1–7.

[ece370176-bib-0029] Hounsome, T. , & Delahay, R. (2005). Birds in the diet of the Eurasian badger *Meles meles*: A review and meta‐analysis. Mammal Review, 35(2), 199–209.

[ece370176-bib-0030] Iriarte, J. A. , Jimenez, J. E. , Contreras, L. C. , & Jaksić, F. M. (1989). Small‐mammal availability and consumption by the fox, *Dusicyon culpaeus*, in central Chilean scrublands. Journal of Mammalogy, 70(3), 641–645.

[ece370176-bib-0032] Jaksic, F. M. (1998). Vertebrate invaders and their ecological impacts in Chile. Biological Conservation, 7(11), 1427–1445.

[ece370176-bib-0033] Jaksic, F. M. (2007). Ecología de Comunidades. Ediciones UC.

[ece370176-bib-0034] Jaksic, F. M. , Jiménez, J. E. , Castro, S. A. , & Feinsinger, P. (1992). Numerical and functional response of predators to a long‐term decline in mammalian prey at a semi‐arid Neotropical site. Oecologia, 89(1), 90–101. 28313400 10.1007/BF00319020

[ece370176-bib-0035] Jaksic, F. M. , Meserve, P. L. , Gutiérrez, J. R. , & Tabilo, E. L. (1993). The components of predation on small mammals in semiarid Chile: Preliminary results. Revista Chilena de Historia Natural, 66, 305–321.

[ece370176-bib-0036] Jaksic, F. M. , Schlatter, R. P. , & Yáñez, J. L. (1980). Feeding ecology of central Chilean foxes, *Dusicyon culpaeus* and *Dusicyon griseus* . Journal of Mammalogy, 61(2), 254–260.

[ece370176-bib-0037] Jiménez, J. E. , & Novaro, A. J. (2004). Culpeo (*Pseudalopex culpaeus*). In C. Sillero‐Zubiri , M. Hoffmann , & D. W. Macdonald (Eds.), Canids: Foxes, wolves, jackals and dogs. Status survey and conservation action plan (pp. 44–49). IUCN/SCC Canid Specialist Group, Gland and Cambridge.

[ece370176-bib-0038] Jiménez, J. E. , Yáñez, J. L. , Tabilo, E. L. , & Jaksic, F. M. (1996). Niche‐complementarity of south American foxes: Reanalysis and test of a hypothesis. Revista Chilena de Historia Natural, 69, 113–123.

[ece370176-bib-0039] Johnson, W. E. , & Franklin, W. L. (1994). Role of body size in the diets of sympatric gray and culpeo foxes. Journal of Mammalogy, 75(1), 163–174.

[ece370176-bib-0040] Kausrud, K. L. , Mysterud, A. , Steen, H. , Vik, J. O. , Østbye, E. , & Cazelles, B. (2008). Linking climate change to lemming cycles. Nature, 456, 93–97. 18987742 10.1038/nature07442

[ece370176-bib-0041] Keith, S. A. , & Bull, J. W. (2017). Animal culture impacts species' capacity to realise climate‐driven range shifts. Ecography, 40(2), 296–304.

[ece370176-bib-0042] Krebs, C. J. (2002). Beyond population regulation and limitation. Wildlife Research, 29(1), 1–10.

[ece370176-bib-0043] Lesmeister, D. B. , Nielsen, C. K. , Schauber, E. M. , & Hellgren, E. C. (2015). Spatial and temporal structure of a mesocarnivore guild in Midwestern North America. Wildlife Monographs, 191(1), 1–61.

[ece370176-bib-0044] Lozano, J. , Guntiñas, M. , Cisneros, R. , Urgilés‐Verdugo, C. , Molina, S. , Zapata‐Ríos, G. , Usiña, J. F. , & Tirira, D. (2021). Lobo de páramo (Lycalopex culpaeus). Libro Rojo de los Mamíferos del Ecuador. Asociación Ecuatoriana de Mastozoología, Fundación Mamíferos y Conservación y Ministerio del Ambiente y Agua del Ecuador.

[ece370176-bib-0045] Lozano, J. , Moleón, M. , & Virgós, E. (2006). Biogeographical patterns in the diet of the wildcat, *Felis silvestris* Schreber, in Eurasia: Factors affecting the trophic diversity. Journal of Biogeography, 33(6), 1076–1085.

[ece370176-bib-0046] Lucherini, M. (2016). Lycalopex culpaeus . IUCN red list of threatened species 2016.

[ece370176-bib-0047] Marquet, P. A. , Contreras, L. C. , Torresmura, J. , Silva, S. I. , & Jaksic, F. M. (1993). Food habits of *Pseudalopex* foxes in the Atacama Desert, pre‐Andean ranges, and the high‐Andean plateau of northernmost Chile. Mammalia, 57(1), 131–135.

[ece370176-bib-0048] Martínez, D. R. , Rau, J. R. , & Jaksic, F. M. (1993). Respuesta numérica y selectividad dietaria de zorros (*Pseudalopex* spp.) ante una reducción de sus presas en el norte de Chile. Revista Chilena de Historia Natural, 66, 195–202.

[ece370176-bib-0049] Medel, R. , & Jaksic, F. M. (1988). Ecología de los cánidos sudamericanos: una revisión. Revista Chilena de Historia Natural, 61(1), 67–79.

[ece370176-bib-0050] Merilä, J. (2012). Evolution in response to climate change: In pursuit of the missing evidence. BioEssays, 34(9), 811–818. 22782862 10.1002/bies.201200054

[ece370176-bib-0051] Meserve, P. L. , Shadrick, E. J. , & Kelt, D. A. (1987). Diets and selectivity of two Chilean predators in the northern semi‐arid zone. Revista Chilena de Historia Natural, 0(1), 93–99.

[ece370176-bib-0052] Monteverde, M. J. , & Piudo, L. (2011). Activity patterns of the culpeo fox (*Lycalopex culpaeus magellanica*) in a non‐hunting area of northwestern Patagonia, Argentina. Mammal Study, 36(3), 119–125.

[ece370176-bib-0053] Newsome, T. M. , Dellinger, J. A. , Pavey, C. R. , Ripple, W. J. , Shores, C. R. , Wirsing, A. J. , & Dickman, C. R. (2015). The ecological effects of providing resource subsidies to predators. Global Ecology and Biogeography, 24(1), 1–11.

[ece370176-bib-0054] Newsome, T. M. , & Ripple, W. J. (2015). A continental scale trophic cascade from wolves through coyotes to foxes. Journal of Animal Ecology, 84, 49–59. 24930631 10.1111/1365-2656.12258

[ece370176-bib-0055] Novaro, A. J. (1997). Pseudalopex culpaeus . Mammalian Species, 558, 1–8.

[ece370176-bib-0056] Novaro, A. J. , Funes, M. C. , & Walker, R. S. (2000). Ecological extinction of native prey of a carnivore assemblage in Argentine Patagonia. Biological Conservation, 92(1), 25–33.

[ece370176-bib-0058] Palacios, R. , Walker, R. S. , & Novaro, A. J. (2012). Differences in diet and trophic interactions of Patagonian carnivores between areas with mostly native or exotic prey. Mammalian Biology, 77(3), 183–189.

[ece370176-bib-0059] Pía, M. V. (2011). Influencia conjunta de la vegetación, asentamientos humanos, caminos y actividades ganaderas sobre la ocurrencia y dieta de los carnívoros tope de Achala (Córdoba, Argentina) . PhD thesis, Universidad Nacional de Córdoba.

[ece370176-bib-0060] Pía, M. V. , López, M. S. , & Novaro, A. J. (2003). Efectos del ganado sobre la ecología trófica del zorro culpeo (*Pseudalopex culpaeus smithersi*) (Carnivora: Canidae) endémico del centro de Argentina. Revista Chilena de Historia Natural, 76(2), 313–321.

[ece370176-bib-0061] Rangel, T. F. , Diniz‐Filho, J. A. F. , & Bini, L. M. (2010). SAM: A comprehensive application for spatial analysis in macroecology. Ecography, 33, 46–50.

[ece370176-bib-0062] Redford, K. H. , & Eisenberg, J. F. (1992). Mammals of the Neotropics, Vol. 2. University of Chicago Press.

[ece370176-bib-0063] Ripple, W. J. , Estes, J. A. , Beschta, R. L. , Wilmers, C. C. , Ritchie, E. G. , Hebblewhite, M. , Berger, J. , Elmhagen, B. , Letnic, M. , Nelson, M. P. , Schmitz, O. J. , Smith, D. W. , Wallach, A. D. , & Wirsing, A. J. (2014). Status and ecological effects of the world's largest carnivores. Science, 343, 1241484. 24408439 10.1126/science.1241484

[ece370176-bib-0064] Ritchie, E. G. , Elmhagen, B. , Glen, A. S. , Letnic, M. , Ludwig, G. , & McDonald, R. A. (2012). Ecosystem restoration with teeth: What role for predators? Trends in Ecology & Evolution, 27, 265–271. 22321653 10.1016/j.tree.2012.01.001

[ece370176-bib-0065] Romo, M. C. (1995). Food habits of the Andean fox (*Pseudalopex culpaeus*) and notes on the moutain cat (*Felis colocolo*) and puma (*Felis concolor*) in the Rio Abiseo National Park, Peru. Mammalia, 59(3), 335–344.

[ece370176-bib-0066] Rosenzweig, M. L. (1995). Species diversity in space and time. Cambridge University Press.

[ece370176-bib-0067] Rubio, A. V. , Alvarado, R. , & Bonacic, C. (2013). Introduced European rabbit as main prey of the native carnivore culpeo fox (*Lycalopex culpaeus*) in disturbed ecosystems of central Chile. Studies on Neotropical Fauna and Environment, 48(2), 89–94.

[ece370176-bib-0068] Sanderson, E. W. , Jaiteh, M. , Levy, M. A. , Redford, K. H. , Wannebo, A. V. , & Woolmer, G. (2002). The human footprint and the last of the wild. Bioscience, 52, 891–904.

[ece370176-bib-0069] Sandom, C. , Faurby, S. , Sandel, B. , & Svenning, J. C. (2014). Global late quaternary megafauna extinctions linked to humans, not climate change. Proceedings of the Royal Society B, 281, 1787. 10.1098/rspb.2013.3254 PMC4071532 24898370

[ece370176-bib-0070] Soe, E. , Davison, J. , Süld, K. , Valdmann, H. , Laurimaa, L. , & Saarma, U. (2017). Europe‐wide biogeographical patterns in the diet of an ecologically and epidemiologically important mesopredator, the red fox *Vulpes vulpes*: A quantitative review. Mammal Review, 47(3), 198–211.

[ece370176-bib-0071] StatSoft Inc . (2011). STATISTICA (data analysis software system), version 10 . https://www.statsoft.com

[ece370176-bib-0073] Underwood, A. J. (1996). Experiments in ecology. Cambridge University Press.

[ece370176-bib-0074] Virgós, E. , & Casanovas, J. G. (1999). Environmental constraints at the edge of a species distribution, the Eurasian badger (*Meles meles* L.): A biogeographic approach. Journal of Biogeography, 26(3), 559–564.

[ece370176-bib-0075] Vulla, E. , Hobson, K. A. , Korsten, M. , Leht, M. , Martin, A. J. , Lind, A. , & Saarma, U. (2009). Carnivory is positively correlated with latitude among omnivorous mammals: Evidence from brown bears, badgers and pine martens. Annales Zoologici Fennici, 46(6), 395–415.

[ece370176-bib-0076] Walker, R. S. , Novaro, A. J. , Perovic, P. , Palacios, R. , Donadio, E. , Lucherini, M. , & López, M. S. (2007). Diets of three species of Andean carnivores in high‐altitude deserts of Argentina. Journal of Mammalogy, 88(2), 519–525.

[ece370176-bib-0077] Weaver, W. , & Shannon, C. E. (1949). The mathematical theory of communication. Illinois University Press.

[ece370176-bib-0078] Zapata, S. C. , Travaini, A. , Delibes, M. , & Martínez‐Peck, R. (2005). Food habits and resource partitioning between grey and culpeo foxes in southeastern Argentine Patagonia. Studies on Neotropical Fauna and Environment, 40(2), 97–103.

[ece370176-bib-0079] Zar, J. H. (2009). Biostatistical analysis. Prentice Hall.

