# Supplementary material for: Diversity and biogeographical patterns in the diet of the culpeo in South America
Source: Ecol Evol. 2024 Aug 13;14(8):e70176. doi: 10.1002/ece3.70176 (PMC11319844; doi:10.1002/ece3.70176)
Supplement: Supplementary file 1 — Data S1. [file ECE3-14-e70176-s001.zip › SupMat1_RFdata.docx]

| **Study** | **Latitude** | **RF Small_mamals** | **RF Carnivorous** | **RF Big_Rodents** | **RF Lagomorphs** | **RF Edentates** | **RF Large_Herbivores** | **RF Birds** | **RF Eggs** | **RF Reptiles/Frogs** | **RF Invertebrates** | **H' index** |
| --- | --- | --- | --- | --- | --- | --- | --- | --- | --- | --- | --- | --- |
| **Achilles. 2007** | 36.45 | 24.72 | 0.57 |  |  |  |  | 15.63 |  | 12.50 | 46.59 | 1.48 |
| **Cornejo & Jiménez. 2001** | 16.32 | 45.73 | 1.38 | 4.13 |  |  |  | 12.40 |  | 2.20 | 34.16 | 1.29 |
| **Ebensperger et al.. 1991** | 33.17 | 15.50 | 0.50 |  | 7.50 |  |  | 5.00 |  | 3.00 | 68.50 | 1.05 |
| **Iriarte et al.. 1989 (Valley)** | 33.23 | 52.31 |  |  | 34.62 |  |  | 0.77 | 0.77 | 5.00 | 6.54 | 1.34 |
| **Iriarte et al.. 1989 (Mountain)** | 33.23 | 68.70 | 0.76 |  | 21.37 |  |  | 0.76 | 1.53 | 4.58 | 2.29 | 1.12 |
| **Jaksic et al.. 1980** | 33.26 | 72.01 |  |  | 18.37 |  |  | 3.21 | 2.04 | 4.37 |  | 1.11 |
| **Johnson & Franklin. 1994** | 51.03 | 19.95 | 0.75 |  | 66.96 |  | 2.99 | 6.73 | 0.62 | 0.37 | 1.62 | 1.16 |
| **Marquet et al.. 1993** | 18.10 | 72.80 |  | 4.80 |  |  | 1.60 | 16.80 | 1.60 | 2.40 |  | 0.72 |
| **Novaro et al.. 2000** | 40.00 | 34.46 |  |  | 30.50 | 1.58 | 26.34 | 5.15 |  | 1.98 |  | 1.70 |
| **Palacios et al.. 2012** | 37.30 | 42.78 | 1.29 | 5.15 | 13.40 | 1.55 | 6.19 | 5.67 |  | 5.67 | 18.30 | 1.72 |
| **Pia et al.. 2003** | 31.34 | 33.96 | 0.57 | 60.57 | 2.08 |  | 0.94 | 1.89 |  |  |  | 0.32 |
| **Romo et al.. 1995** | 7.50 | 51.56 |  | 16.41 |  |  |  | 17.19 |  | 7.81 | 7.03 | 1.28 |
| **Rubio et al.. 2013 (Site 1)** | 33.41 | 14.29 |  |  | 74.15 |  |  | 6.80 | 4.08 |  | 0.68 | 0.96 |
| **Rubio et al.. 2013 (Site 2)** | 33.41 | 31.76 |  |  | 58.82 |  |  | 5.88 | 1.76 | 0.59 | 1.18 | 1.08 |
| **Walker et al.. 2007** | 25.60 | 39.78 | 1.20 | 3.28 | 6.89 |  | 15.85 | 17.16 | 0.33 | 3.93 | 11.58 | 1.66 |
| **Zapata et al.. 2005** | 47.39 | 35.09 | 1.75 |  | 30.41 | 6.73 | 6.43 | 9.94 | 1.46 | 1.17 | 7.02 | 1.88 |
| **Guntiñas et al.. 2017** | 4.60 | 19.80 | 7.43 | 9.16 | 6.19 | 0.99 | 53.70 | 2.23 |  |  | 0.50 | 1.57 |
| **Berg. 2009** | 36.10 | 30.30 |  | 12.12 | 4.55 | 4.55 | 22.73 | 1.52 |  |  | 24.24 | 1.53 |
| **Monteverde et al.. 2011** | 40.08 | 72.00 |  |  | 16 |  | 2.00 | 4.00 |  |  | 6.00 | 0.36 |
