# Supplementary material for: Diversity and biogeographical patterns in the diet of the culpeo in South America
Source: Ecol Evol. 2024 Aug 13;14(8):e70176. doi: 10.1002/ece3.70176 (PMC11319844; doi:10.1002/ece3.70176)
Supplement: Supplementary file 1 — Data S1. [file ECE3-14-e70176-s001.zip › SupMat2_Species_List.docx]

List of species and other food items belonging to culpeo’s diet reported in this study.

**Small rodents Big rodents Mustelids Birds Plant species**

| *Abrocoma bennetti Abrocoma cinerea Abrothrix longipilis* |  | *Cavia tschudii Caviidae spp. Chinchilla chinchilla* |  | *Galictis cuja*  **Mephitids** |  | *Bolborhynchus spp. Chloephaga picta Diglossa spp.* |  | *Berberis microphylla Berberis spp.*  *Cryptocarya alba* |
| --- | --- | --- | --- | --- | --- | --- | --- | --- |
| *Abrothrix olivaceus* |  | *Ctenomys opimus* |  |  |  | *Eudromia elegans* |  | *Ephedra americana* |
| *Abrothrix andinus* |  | *Cuniculus paca* |  | *Conepatus humboldtii* |  | Fringillidae |  | *Greigia sphacelata* |
| *Aconaemys fuscus* |  | *Dasyprocta punctata* |  | *Conepatus semistriatus* |  | Fumariidae |  | *Mitraria coccinea* |
| *Akodon albiventer Abrothrix andinus* |  | *Dolichotis patagonum Lagidium peruanum* |  | **Procyonids** |  | Passeriformes  *Phoenicopterus chilensis* |  | *Prosopis pallida Prunus domestica* |
| *Akodon spp.* |  | *Lagidium viscacia* |  | *Nasuella olivacea* |  | *Pteroptochos tarnii* |  | *Rubus geoides* |
| *Andinomys edax* |  | *Lagostomus maximus* |  |  |  | *Rhea pennata* |  | *Schinus molle* |
| *Auliscomys boliviensis* |  | *Microcavia australis* |  | **Felids** |  | *Scytalopus spp.* |  | *Ugni molinae* |
| *Auliscomys pictus* |  | *Sciurus spp.* |  |  |  |  |  | *Vaccinium spp.* |
| *Chelemys macronyx* |  |  |  | *Puma concolor* |  | **Reptiles** |  |  |
| *Cricetidos spp.* |  | **Lagomorphs** |  | *Felis catus* |  |  |  |  |
| *Ctenomys spp.* |  |  |  |  |  | *Liolaemus spp.* |  |  |
| *Eligmodontia typus* |  | *Lepus europaeus* |  |  |  | *Liolaemus chiliensis* |  |  |
| *Loxodontomys micropus* |  | *Oryctolagus cuniculus* |  | **Procyonids** |  | *Philodryas chamissonis* |  |  |
| *Mus musculus* |  | *Sylvilagus brasiliensis* |  | *Nasuella olivacea* |  | *Tachymenis peruviana* |  |  |
| *Neotomys spp.* |  |  |  |  |  | Lizards |  |  |
| *Octodon bridgesii* |  | **Marsupials** |  | **Camelids** |  | **Amphibians** (frogs) |  |  |
| *Octodon degus* |  |  |  |  |  |  |  |  |
| *Octodontomys gliroides* |  | *Thylamys pusilla* |  | *Lama guanicoe* |  | **Other food items** |  |  |
| *Oligoryzomys longicaudatus* |  | *Caenolestes caniventer* |  | *Lama glama* |  |  |  |  |
| *Oryzomys spp.* |  | *Caenolestes fuliginosus* |  | *Vicugna vicugna* |  | Livestock (*Ovis* spp.) |  |  |
| *Oxymycterus* |  | *Caenolestes spp.* |  |  |  | Eggs |  |  |
| *Phyllotis spp.* |  | *Didelphis spp.* |  | **Cervids** |  | Carrion |  |  |
| *Phyllotis darwini* |  | *Dromiciops gliroides* |  |  |  | Fishes |  |  |
| *Phyllotis limatus* |  | *Marmosa robinsoni* |  | *Mazama rufina* |  | Rubbish |  |  |
| *Phyllotis magister* |  | *Marmosa spp.* |  | *Pudu mephistophiles* |  |  |  |  |
| *Phyllotis xanthopygus* |  | *Thylamys elegans* |  |  |  |  |  |  |
| *Rattus rattus* |  |  |  | **Edentates** |  |  |  |  |
| *Reithrodon physoles* |  | **Soricids**  **Soricids** |  | *Dasypus novemcinctus* |  |  |  |  |
| *Spalacopus cyanus* |  | *Cryptotis montivaga* |  | *Zaedyus pichiy* |  |  |  |  |
| *Thomasomys spp.* |  |  |  | *Chaetophractus spp.* |  |  |  |  |
