# Supplementary material for: Diversity and biogeographical patterns in the diet of the culpeo in South America
Source: Ecol Evol. 2024 Aug 13;14(8):e70176. doi: 10.1002/ece3.70176 (PMC11319844; doi:10.1002/ece3.70176)
Supplement: Supplementary file 1 — Data S1. [file ECE3-14-e70176-s001.zip › Supplement.docx]

**SM 1.** Culpeo diet as described in the reviewed studies. Diet data are expressed as relative frequency (RF) of each prey group in relation to the minimum total number of identified prey. Latitude of each study area and trophic diversity values (Shannon-Wiener index, H’) are also shown.

| **Study** | **Latitude** | **RF Small_mamals** | **RF Carnivorous** | **RF Big_Rodents** | **RF Lagomorphs** | **RF Edentates** | **RF Large_Herbivores** | **RF Birds** | **RF Eggs** | **RF Reptiles/Frogs** | **RF Invertebrates** | **H' index** |
| --- | --- | --- | --- | --- | --- | --- | --- | --- | --- | --- | --- | --- |
| **Achilles. 2007** | 36.45 | 24.72 | 0.57 |  |  |  |  | 15.63 |  | 12.50 | 46.59 | 1.48 |
| **Cornejo & Jiménez. 2001** | 16.32 | 45.73 | 1.38 | 4.13 |  |  |  | 12.40 |  | 2.20 | 34.16 | 1.29 |
| **Ebensperger et al.. 1991** | 33.17 | 15.50 | 0.50 |  | 7.50 |  |  | 5.00 |  | 3.00 | 68.50 | 1.05 |
| **Iriarte et al.. 1989 (Valley)** | 33.23 | 52.31 |  |  | 34.62 |  |  | 0.77 | 0.77 | 5.00 | 6.54 | 1.34 |
| **Iriarte et al.. 1989 (Mountain)** | 33.23 | 68.70 | 0.76 |  | 21.37 |  |  | 0.76 | 1.53 | 4.58 | 2.29 | 1.12 |
| **Jaksic et al.. 1980** | 33.26 | 72.01 |  |  | 18.37 |  |  | 3.21 | 2.04 | 4.37 |  | 1.11 |
| **Johnson & Franklin. 1994** | 51.03 | 19.95 | 0.75 |  | 66.96 |  | 2.99 | 6.73 | 0.62 | 0.37 | 1.62 | 1.16 |
| **Marquet et al.. 1993** | 18.10 | 72.80 |  | 4.80 |  |  | 1.60 | 16.80 | 1.60 | 2.40 |  | 0.72 |
| **Novaro et al.. 2000** | 40.00 | 34.46 |  |  | 30.50 | 1.58 | 26.34 | 5.15 |  | 1.98 |  | 1.70 |
| **Palacios et al.. 2012** | 37.30 | 42.78 | 1.29 | 5.15 | 13.40 | 1.55 | 6.19 | 5.67 |  | 5.67 | 18.30 | 1.72 |
| **Pia et al.. 2003** | 31.34 | 33.96 | 0.57 | 60.57 | 2.08 |  | 0.94 | 1.89 |  |  |  | 0.32 |
| **Romo et al.. 1995** | 7.50 | 51.56 |  | 16.41 |  |  |  | 17.19 |  | 7.81 | 7.03 | 1.28 |
| **Rubio et al.. 2013 (Site 1)** | 33.41 | 14.29 |  |  | 74.15 |  |  | 6.80 | 4.08 |  | 0.68 | 0.96 |
| **Rubio et al.. 2013 (Site 2)** | 33.41 | 31.76 |  |  | 58.82 |  |  | 5.88 | 1.76 | 0.59 | 1.18 | 1.08 |
| **Walker et al.. 2007** | 25.60 | 39.78 | 1.20 | 3.28 | 6.89 |  | 15.85 | 17.16 | 0.33 | 3.93 | 11.58 | 1.66 |
| **Zapata et al.. 2005** | 47.39 | 35.09 | 1.75 |  | 30.41 | 6.73 | 6.43 | 9.94 | 1.46 | 1.17 | 7.02 | 1.88 |
| **Guntiñas et al.. 2017** | 4.60 | 19.80 | 7.43 | 9.16 | 6.19 | 0.99 | 53.70 | 2.23 |  |  | 0.50 | 1.57 |
| **Berg. 2009** | 36.10 | 30.30 |  | 12.12 | 4.55 | 4.55 | 22.73 | 1.52 |  |  | 24.24 | 1.53 |
| **Monteverde et al.. 2011** | 40.08 | 72.00 |  |  | 16 |  | 2.00 | 4.00 |  |  | 6.00 | 0.36 |

**SM II.** List of species and other food items belonging to culpeo diet reported in the reviewed studies.

**Small rodents Big rodents Mustelids Birds Plant species**

| *Abrocoma bennetti Abrocoma cinerea Abrothrix longipilis* |  | *Cavia tschudii Caviidae spp. Chinchilla chinchilla* |  | *Galictis cuja*  **Mephitids** |  | *Bolborhynchus spp. Chloephaga picta Diglossa spp.* |  | *Berberis microphylla Berberis spp.*  *Cryptocarya alba* |
| --- | --- | --- | --- | --- | --- | --- | --- | --- |
| *Abrothrix olivaceus* |  | *Ctenomys opimus* |  |  |  | *Eudromia elegans* |  | *Ephedra americana* |
| *Abrothrix andinus* |  | *Cuniculus paca* |  | *Conepatus humboldtii* |  | Fringillidae |  | *Greigia sphacelata* |
| *Aconaemys fuscus* |  | *Dasyprocta punctata* |  | *Conepatus semistriatus* |  | Fumariidae |  | *Mitraria coccinea* |
| *Akodon albiventer Abrothrix andinus* |  | *Dolichotis patagonum Lagidium peruanum* |  | **Procyonids** |  | Passeriformes  *Phoenicopterus chilensis* |  | *Prosopis pallida Prunus domestica* |
| *Akodon spp.* |  | *Lagidium viscacia* |  | *Nasuella olivacea* |  | *Pteroptochos tarnii* |  | *Rubus geoides* |
| *Andinomys edax* |  | *Lagostomus maximus* |  |  |  | *Rhea pennata* |  | *Schinus molle* |
| *Auliscomys boliviensis* |  | *Microcavia australis* |  | **Felids** |  | *Scytalopus spp.* |  | *Ugni molinae* |
| *Auliscomys pictus* |  | *Sciurus spp.* |  |  |  |  |  | *Vaccinium spp.* |
| *Chelemys macronyx* |  |  |  | *Puma concolor* |  | **Reptiles** |  |  |
| *Cricetidos spp.* |  | **Lagomorphs** |  | *Felis catus* |  |  |  |  |
| *Ctenomys spp.* |  |  |  |  |  | *Liolaemus spp.* |  |  |
| *Eligmodontia typus* |  | *Lepus europaeus* |  |  |  | *Liolaemus chiliensis* |  |  |
| *Loxodontomys micropus* |  | *Oryctolagus cuniculus* |  | **Procyonids** |  | *Philodryas chamissonis* |  |  |
| *Mus musculus* |  | *Sylvilagus brasiliensis* |  | *Nasuella olivacea* |  | *Tachymenis peruviana* |  |  |
| *Neotomys spp.* |  |  |  |  |  | Lizards |  |  |
| *Octodon bridgesii* |  | **Marsupials** |  | **Camelids** |  | **Amphibians** (Frogs) |  |  |
| *Octodon degus* |  |  |  |  |  |  |  |  |
| *Octodontomys gliroides* |  | *Thylamys pusilla* |  | *Lama guanicoe* |  | **Other food items** |  |  |
| *Oligoryzomys longicaudatus* |  | *Caenolestes caniventer* |  | *Lama glama* |  |  |  |  |
| *Oryzomys spp.* |  | *Caenolestes fuliginosus* |  | *Vicugna vicugna* |  | Livestock (*Ovis* spp.) |  |  |
| *Oxymycterus* |  | *Caenolestes spp.* |  |  |  | Eggs |  |  |
| *Phyllotis spp.* |  | *Didelphis spp.* |  | **Cervids** |  | Carrion |  |  |
| *Phyllotis darwini* |  | *Dromiciops gliroides* |  |  |  | Fishes |  |  |
| *Phyllotis limatus* |  | *Marmosa robinsoni* |  | *Mazama rufina* |  | Rubbish |  |  |
| *Phyllotis magister* |  | *Marmosa spp.* |  | *Pudu mephistophiles* |  |  |  |  |
| *Phyllotis xanthopygus* |  | *Thylamys elegans* |  |  |  |  |  |  |
| *Rattus rattus* |  |  |  | **Edentates** |  |  |  |  |
| *Reithrodon physoles* |  | **Soricids**  **Soricids** |  | *Dasypus novemcinctus* |  |  |  |  |
| *Spalacopus cyanus* |  | *Cryptotis montivaga* |  | *Zaedyus pichiy* |  |  |  |  |
| *Thomasomys spp.* |  |  |  | *Chaetophractus spp.* |  |  |  |  |
