# Supplementary material for: Diversity and biogeographical patterns in the diet of the culpeo in South America
Source: Ecol Evol. 2024 Aug 13;14(8):e70176. doi: 10.1002/ece3.70176 (PMC11319844; doi:10.1002/ece3.70176)
Supplement: Supplementary file 1 — Data S1. [file ECE3-14-e70176-s001.zip › Translated Abstract not for Review.pdf]

RESUMEN

Este artículo describe los patrones tróficos del culpeo (o zorro andino) a escala biogeográfica. También se analiza la influencia de los lagomorfos exóticos en su dieta y exploramos las diferencias entre las subespecies de culpeo. Seleccionamos 17 estudios de dieta comparables entre sí, que incluyen 19 evaluaciones independientes de la dieta. A continuación, estandarizamos los valores de los diferentes componentes de la dieta de estos estudios y calculamos la frecuencia relativa de aparición (RF) de los diez principales grupos tróficos. Además, calculamos el índice de diversidad trófica Shannon-Wiener H' para cada muestra independiente.

Los resultados mostraron que los pequeños mamíferos (41%), los lagomorfos (21%), los invertebrados (12,4%) y los grandes herbívoros (7,3%) fueron los grupos más consumidos por el culpeo. Un análisis de factores de todos los grupos tróficos arrojó cuatro factores ortogonales que se utilizaron como variables respuesta en relación a un conjunto de predictores ambientales. La altitud se correlacionó con la mayoría de los factores (grupos tróficos). Los lagomorfos exóticos fueron consumidos en tierras bajas, en latitudes más altas y en regiones que mostraron valores elevados del índice de huella humana, enriqueciendo en esas zonas el espectro trófico del culpeo. No hubo diferencias en la dieta entre las dos principales subespecies de culpeo analizadas, *L.c. culpaeus* y *L.c. andinus.*

Por último, los mejores modelos explicativos (GLM) de la diversidad trófica seleccionados, utilizando el criterio de información de Akaike (AIC), mostraron que las dietas más diversas del culpeo son las compuestas por grandes herbívoros, edentados, especies carnívoras, aves y herpetos (esto es, anfibios y reptiles). La diversidad trófica fue baja en las zonas lluviosas y donde los grandes roedores dominaron la dieta. Ni la latitud ni la altitud parecieron tener efecto sobre la diversidad trófica de los culpeos, ya que no fueron retenidas por los modelos finales.
